# Integrative analysis of gene expression and methylation data for breast cancer cell lines

**DOI:** 10.1186/s13040-018-0174-8

**Published:** 2018-06-25

**Authors:** Catherine Li, Juyon Lee, Jessica Ding, Shuying Sun

**Affiliations:** 1 Westwood High School, Austin, Texas USA; 2 Korea International School Pangyo Campus, Seongnam, South Korea; 3 Liberal Arts and Science Academy, Austin, Texas USA; 4 Department of Mathematics, Texas State University, San Marcos, TX USA

**Keywords:** DNA methylation, Gene expression, Data integration, Pathway analysis, Breast cancer

## Abstract

**Background:**

The deadly costs of cancer and necessity for an accurate method of early cancer detection have demanded the identification of genetic and epigenetic factors associated with cancer. DNA methylation, an epigenetic event, plays an important role in cancer susceptibility. In this paper, we use DNA methylation and gene expression data integration and pathway analysis to further explore and understand the complex relationship between methylation and gene expression.

**Results:**

Through linear modeling and analysis of variance, we obtain genes that show a significant correlation between methylation and gene expression. We then examine the functions and relationships of these genes using bioinformatic tools and databases. In particular, using ConsensusPathDB, we analyze the networks of statistically significant genes to identify hub genes, genes with a large number of links to other genes. We identify eight major hub genes, all in strong association with cancer susceptibility. Through further analysis of the function, gene expression level, and methylation level of these hub genes, we conclude that they are novel potential biomarkers for breast cancer.

**Conclusions:**

Our findings have various implications for cancer screening, early detection methods, and potential novel treatments for cancer. Researchers can also use our results to develop more effective methods for cancer study.

**Electronic supplementary material:**

The online version of this article (10.1186/s13040-018-0174-8) contains supplementary material, which is available to authorized users.

## Background

Even after decades of research, cancer is still the second biggest killer in the United States according to statistics provided by the National Institutes of Health (NIH). According to statistics from the NIH, by 2020, medical expenditures for cancer are projected to be at least $158 billion, excluding inflation. The NIH further reports that the most expensive cancer to treat is breast cancer with $16.5 billion spent in 2010 alone. These statistics not only call for more effective and cheaper treatments, but also accurate early detection of cancer. Currently, medical imaging is the most common form of cancer screening. However, these forms of testing are expensive and rarely administered. Thus, a more universal, cost-efficient, and effective way of early detection is necessary and the identification of genetic and epigenetic factors associated with cancer is imperative. In this project, we will study DNA methylation, an important epigenetic factor, and its regulatory roles on gene expression using data from breast cancer cell lines.

To begin, we will briefly review a few concepts in genetics and epigenetics. A *cell line* is a cultured cell type that can be reproduced indefinitely. A *gene* is a region of a chromosome that codes for a particular protein. *DNA methylation* is an epigenetic event. In mature mammalians, methylation occurs when a methyl group (-CH_3_) bonds to a cytosine in the dinucleotide pair CpG. A *CpG, or CG, site* is a cytosine base connected to a guanine base by a phosphate bond. These CpG sites are not evenly distributed throughout a genome, but rather clump together in particular regions, which are called *CpG islands*. Methylated CpG islands located in the promoters of genes are important for researchers to study, because methylation that occurs in promoter regions can inhibit transcription factors from binding to promoters, which prevents transcription and turns a gene off. *Hypomethylation* is a low level or loss of methylation while *hypermethylation* is a high level or gain of methylation [1]. Additionally, *Estrogen Receptor (ER) status* and *tumor subtype* are closely related to gene expression levels [2]. Estrogen receptors are common hormone receptors and the two ER statuses are *ER+* and *ER-*. *ER+* refers to a breast cancer that is dependent on estrogen to grow, while *ER-* refers to a breast cancer that is not dependent on estrogen stimulation. The three tumor subtypes we studied are *Luminal, Basal A*, and *Basal B*, as defined by Neve, et al. [2]. *Luminal* tumors are ERBB3 and ESR1 positive, while *Basal* tumors are ESR1 negative and CAV1 positive. *Basal* tumors can be further characterized into two different subtypes: A (KRT5 and KRT14 positive) and B (VIM positive), where ERBB3, ESR1, CAV1, KRT5, KRT14 and VIM are all crucial hormone receptors. *Basal B* has been shown to represent the “triple-negative” tumor, the most aggressive tumor that is also the hardest to treat.

DNA methylation is known to be associated with gene imprinting, X-chromosome inactivation, normal development, the loss of transcription, and the suppression of parasitic DNA sequences to list a few [3–5]. This is because CpG islands often overlap or are associated with transcription start sites (TSS) of genes. Therefore, methylation of these CpG islands can result in the loss of transcription of important genes. Additionally, transition mutations can occur if a methylated cytosine undergoes deamination and transforms into thymine, which essentially changes the DNA sequence. Thus, it is clear that DNA methylation plays a significant role in gene expression through mutations and gene silencing. These changes in gene expression can have an enormous impact, ranging from abnormal cell differentiation to human diseases and birth defects, making DNA methylation an important field of research. DNA methylation is also linked to cancer development [5]. Methylation on promoters of tumor suppressor genes, cell cycle regulation genes, etc. can turn these genes off and cause genetic instability, a defining feature of cancer. Without active transcription of these genes, the cell cycle may be dysregulated and the body becomes more susceptible to tumors. Using epigenetic data and studying DNA methylation can help identify potential genetic indicators of cancer and allow for prevention and early detection, which is crucial to cancer treatment.

Data integration is important and challenging [6]. Currently, there has been some work done on studying various methylation patterns and linking significantly methylated genes to known cancer-related genes [7]. Sun et al. [8] integrated gene expression and methylation data to identify overexpressed genes in different ER statuses as well as identified differentially expressed and methylated genes. They also discovered a general pattern of differential CpG island methylation. Li et al. [9] did a similar analysis with ovarian cancer cell lines, and their program sMBPLS showed there were interesting relationships between methylation and gene expression without studying the relationship in depth. Louhimo et al.’s [10] CNAmet software package prioritizes putative cancer driver genes by integrating copy number, DNA methylation, and expression data. Their analysis gives information on methylation alteration induced expression, copy number aberration induced expression, and adjacent *p*-values.

In this paper, we focus on studying the effect of methylation on gene expression. However, we also include ER status and tumor subtype in our model to account for the gene expression variation due to these factors. We then use network analysis to study the relationships among genes that demonstrate significant methylation and expression correlation, in order to identify potential biomarkers.

## Methodology

We plan to integrate DNA methylation and gene expression data for breast cancer cell lines to determine which methylated genes affect gene expression and lead to abnormal genetic functions. This integration analysis will help us discover the complex relationship and interactions between methylation and gene expression and identify potential novel biomarkers for breast cancer. Our datasets consist of gene expression and methylation microarray data for 40 breast cancer cell lines [2, 11] and a list of these cell lines with their corresponding tumor subtype and ER status [2].

Gene expression microarray datasets are generated using the Affymetrix high-density oligonucleotide array human HG-U133A chip, which produced CEL files [2]. The Robust Multichip Average (RMA) algorithm is then used to summarize the gene expression level for each probeset [12]. Gene expression levels are calculated as the log abundance of each probeset and should be positive. In each cell line, gene expression levels range from roughly 2 to 13.5.

Methylation microarray datasets are generated using the differential methylation hybridization (DMH) protocol [13–15]. This protocol is implemented using the Agilent 244K two-color array. For Agilent two-color array, samples are labeled using two different fluorophores (usually a red fluorescent dye, and a green fluorescent dye) and hybridized simultaneously onto each probe (or spot) of the array (or chip). The arrays are then laser-scanned and images are processed to obtain the data for analysis. In general, the log ratio of red over green at each probe is used as a measurement. According to the previous research work, it is better to use the internal control probes to preprocess the data [11]. We then use the internal control probes to conduct the within-array normalization for each array and obtain the M-value, which is the base two log ratio of red over green intensity, log_2_(R/G). The M value is used as the observed methylation signal at each probe. In theory, the methylation level range is (−∞, +∞); in practice, for each sample, the methylation levels of all probes roughly range from − 4 to 4, with some variation across samples. All the preprocessing steps for the methylation microarray data are conducted using the Bioconductor package “limma” [16].

Datasets used in this paper are all publicly available. After the above preprocessing steps, we obtain two datasets. One dataset contains the gene expression levels of the 40 cell lines for each of 22,283 probesets, obtained from Neve et al.’s paper [2]. Another dataset contains the methylation levels of 243,504 probes for each of the same 40 cell lines, from Sun et al.’s paper [11]. Next we will outline each step of our project.

1. Using linear models *y*_*i*_ = *β*_0_ + *β*_1_*x*_*i*_ + *β*_2_*ER*_*i*_ + *β*_3_*sub*_*i*_ + *ε*_*i*_, where *i* is the index of the 40 cell lines, *y*_*i*_ is gene expression level, *x*_*i*_ is methylation level, *ER*_*i*_ is ER status, *sub*_*i*_ is tumor subtype, *β*_*i*_’s are coefficients, and *ε*_*i*_ is the error that follows a normal distribution with a mean of 0 and variance *σ*^2^ (i.e., *ε*_*i*_ ∼ N(0, *σ*^2^)), we will perform analysis of variance (ANOVA) and report the methylation *p* value and coefficient. ER status and tumor subtype are included to account for the gene expression variation due to these factors. Thus, we can identify the genes that show significant correlation between gene expression and methylation and with other factors (e.g., ER status and tumor subtype) well accounted for.
2. Using linear models and analysis of variance, we summarize each gene and, using different criteria based upon the gene length, extract ones that demonstrate a significant correlation between gene expression and methylation.
3. Finally, we study the relationship among genes extracted through the above analyses. This bioinformatic study will be done through genetic pathway and network analysis. We will also study the function of selected genes using GeneCards [17] and the Molecular Signatures Database, which is designed based on Gene Set Enrichment Analysis [18].

The above analysis will help researchers gain a much deeper understanding of the interactions of gene expression and methylation in breast cancer cells. This understanding will help us elucidate the function of DNA methylation on an entire genome. It can also allow us to identify novel genetic and epigenetic biomarkers, which may pave the way for new and better diagnosis and treatment of breast cancer in the near future.

All of our datasets are analyzed and processed using code written in R, a statistical language. Additionally, we create induced network modules using the ConsensusPathDB (CPDB) software [19–21], in order to visualize genetic pathways that shows the interactions among genes. CPDB takes a list of genes and connects them through different types of interactions. We consider only high-confidence binary protein interactions, genetic interactions, and regulatory interactions to ensure accuracy. We also allow for intermediate nodes that CPDB includes, in order to further examine the relationships among genes.

## Results

### Linear models and analysis of variance

We utilize the linear model function (*lm*) in R to model gene expression, methylation, ER status, and tumor subtype. Specifically, our model is *y*_*i*_ = *β*_0_ + *β*_1_*x*_*i*_ + *β*_2_*ER*_*i*_ + *β*_3_*sub*_*i*_ + *ε*_*i*_, where *i* is the index of 40 breast cancer cell lines (*i* = 1, ..., 40), *y*_*i*_ is gene expression level, *x*_*i*_ is methylation level, *ER*_*i*_ is ER status, *sub*_*i*_ is tumor subtype, *β*_*i*_’s are coefficients, and *ε*_*i*_ is the error that follows a normal distribution with a mean of 0 and variance *σ*^2^ (i.e., *ε*_*i*_ ∼ N(0, *σ*^2^)). It is important to note that our method uses a gene-wise approach by integrating information from all probesets (from gene expression microarray data) and probes (from methylation microarray data). Because a single gene can correspond to multiple probesets (transcripts for gene expression array data) and probes (DNA sequences for methylation array data), we use *m* to denote the number of probesets associated with a particular gene in the gene expression data, and *n* as the number of probes associated with the same gene in the methylation data. Then, *m* × *n* would be the total number of distinct expression probeset and methylation probe pairs associated with a gene, and *j*, from our model, refers to the index of all *m* × *n* pairs (*j* = 1, 2, ..., *m* × *n*). We investigate which genes’ expression levels are associated with methylation by fitting linear models to all *m* × *n* pairs.

Using the probability (*p*-value) generated from the ANOVA output of each linear model, we determine if the methylation coefficient (*β*_1_) is significantly different from zero. A significantly positive coefficient indicates that increased methylation levels are associated with high gene expression. A significantly negative coefficient means that high methylation levels are associated with low gene expression levels. Using the *p* value and coefficient, we identify genes with a positive correlation between gene expression and methylation and those with a negative correlation. To make sure our results are biologically significant, we look for large correlations and thus extract genes with methylation coefficients |*methy.coeff*| *>* 0*.*5 and an even more stringent condition |*methy.coeff*| *>* 1. We then extract genes fitting both conditions (i.e., *p* value and methylation coefficient). Our results are summarized in Table 1. “Pairs” refers to the number of probeset and probe (*m* × *n*) pairings that qualify under each condition; “Genes”, on the other hand, means the number of unique genes that meet each specification. The number of genes is less than the number of pairs because a unique gene may correspond to several probeset and probe pairs.

**Table 1:** Number of probeset and probe pairings and number of unique genes per specification

	methy.coeff*>* 0.5 & *p <* 0.05	methy.coeff*<*(− 0.5) & *p <* 0.05	methy.coeff*>* 1 & *p <* 0.05	methy.coeff*<*(− 1) & *p <* 0.05
Pairs	1582	1319	261	199
Genes	829	779	156	134

### Significant genes

In order to further analyze these genes, we use the same linear modeling and analysis of variance described above and summarize each gene by examining all of the *m* × *n* pairs and determining how many are significant. Looking at the number of probesets and probes each unique gene corresponds to, there is a large variation among the genes. Therefore, we label a gene as “long” (i.e., a long gene) if it has more than two corresponding probesets in the gene expression data or more than 20 corresponding probes in the methylation data. Otherwise, we call it a “short” gene. Long genes have more pairs of probesets and probes than short genes, so it is not proper to use the same criteria for long and short genes. Distinguishing between long and short allows us to apply more stringent cut-off values on the short genes and more lenient cut-off values when analyzing the long genes. Furthermore, we call a gene’s correlation positive if more than half of the probeset and probe pairs have a positive methylation coefficient. Similarly, the correlation is negative if more than half of the pairs have a negative methylation coefficient. If both of these conditions are false, we conclude that the gene has no correlation.

Using a methylation *p <* 0.05, we determine how many *m* × *n* pairs show statistically significant gene expression and methylation correlation. Then, we obtain long genes with more than 15% significant pairs, and extract short genes with more than 30% significant pairs, out of the total number of *m* × *n* pairs. This yields 273 long genes and 213 short genes, which are shown in Table 2. Then, out of the 273 long genes, 165 have positive correlation between methylation and gene expression, and 95 have negative correlation. Of the 213 significant short genes, 89 have positive correlation and 87 have negative correlation, see Table 2. In order to further investigate the positively correlated genes and the negatively correlated genes, we combine the 165 positively correlated long genes with the 89 positively correlated short genes to create a gene list of 254 genes with positive correlation. Similarly, combining the 95 negatively correlated long genes with the 87 negatively correlated short genes creates a gene list of 182 genes with negative correlation.

**Table 2:** Number of significant long and short genes per specification

Long	% sig pairs *>* 15%	% sig pairs *>* 15% and positive correlation	% sig pairs *>* 15% and negative correlation
# of Genes	273	165	95
Short	% sig pairs *>* 30%	% sig pairs *>* 30% and positive correlation	% sig pairs *>* 30% and negative correlation
# of Genes	213	89	87

To ensure the genes of interest have a strong and significant correlation between methylation and expression, we further study genes reported in both Table 1 and Table 2. Specifically, we find the overlap of genes in both the list of 829 (i.e., genes with a methylation coefficient greater than 0.5 and a methylation *p* value less than 0.05, shown in Table 1) and the list of 254 (i.e., 165 + 89 significantly methylated long and short genes with a positive correlation between methylation and gene expression, shown in Table 2). This gives us 109 genes with significant methylation levels and a positive correlation between methylation and gene expression. Applying the same method to the list of 779 (i.e., genes with a methylation coefficient less than − 0.5 and a methylation p value less than 0.05, shown in Table 1) and the list of 182 (i.e., 95 + 87 genes significantly methylated long and short genes with a negative correlation, shown in Table 2), we obtain 86 significantly methylated genes with a negative correlation between methylation and expression. These results are summarized in Table 3.

**Table 3:** Number of genes with significant correlation

	positive correlation	negative correlation
Significant long/short genes	254	182
	*methy.coeff >* 0*.*5	*methy.coeff <* (−0*.*5)
Methylation *p <* 0.05	829	779
Genes in both lists	109	86

### Gene family analysis using MSigDB

After obtaining these lists of genes with significant correlation between methylation and gene expression, we employ the Molecular Signatures Database (MSigDB), a collection of annotated gene sets [18] to investigate the function of each of these genes. We enter both the 109 positively correlated genes and the 86 negatively correlated genes (195 total) into MSigDB and group the genes by gene family. A gene family is defined as any collection of proteins that share a common feature, such as homology or biochemical activity [18]. The results of the categorization are shown in Table 4.

**Table 4:** Gene families for the 195 significant genes

	cytokines and growth factors	transcription factors	homeodomain proteins	cell differentiation markers	translocated cancer genes	oncogenes	tumor suppressors
tumor suppressors	0	2	0	0	0	0	3
oncogenes	0	2	0	0	7	8	
translocated cancer genes	0	2	0	0	7		
cell differentiation markers	1	0	0	8			
homeodomain proteins	0	2	7				
transcription factors	0	27					
cytokines and growth factors	6						

As can be seen from Tables 4, 27 of the 195 genes (28*.*42%) are transcription factors and 8 are oncogenes. Transcription factors are molecules that determine gene activities by regulating transcription. They are crucial to normal cell function and can have important roles in cancer if they affect genes involved with the cell cycle or cell division. Oncogenes are genes that can potentially induce cancer. Before being mutated, these genes are involved with cell differentiation and proliferation, as well as cell growth. Then, a mutation to these genes can change the gene function, leading to uncontrollable cell reproduction and forming a cancerous tumor. Because these transcription factors and oncogenes can play such a significant role in cancer development, we further study the interactions of the genes in detail by utilizing the CPDB software [19–21] to generate induced network modules.

### Pathway analysis

To determine the relationship and interactions among these identified significant genes, we input the gene names into CPDB. With CPDB, we consider only high-confidence binary protein interactions, genetic interactions, and gene regulatory interactions, and we also allow intermediate nodes. We choose these options in order to only display highly accurate and important interactions, which will allow us to draw the most relevant conclusions. The nodes labeled with magenta text are intermediate nodes, which are genes that CPDB includes in the network and not on our input gene list.

**Fig. 1:**
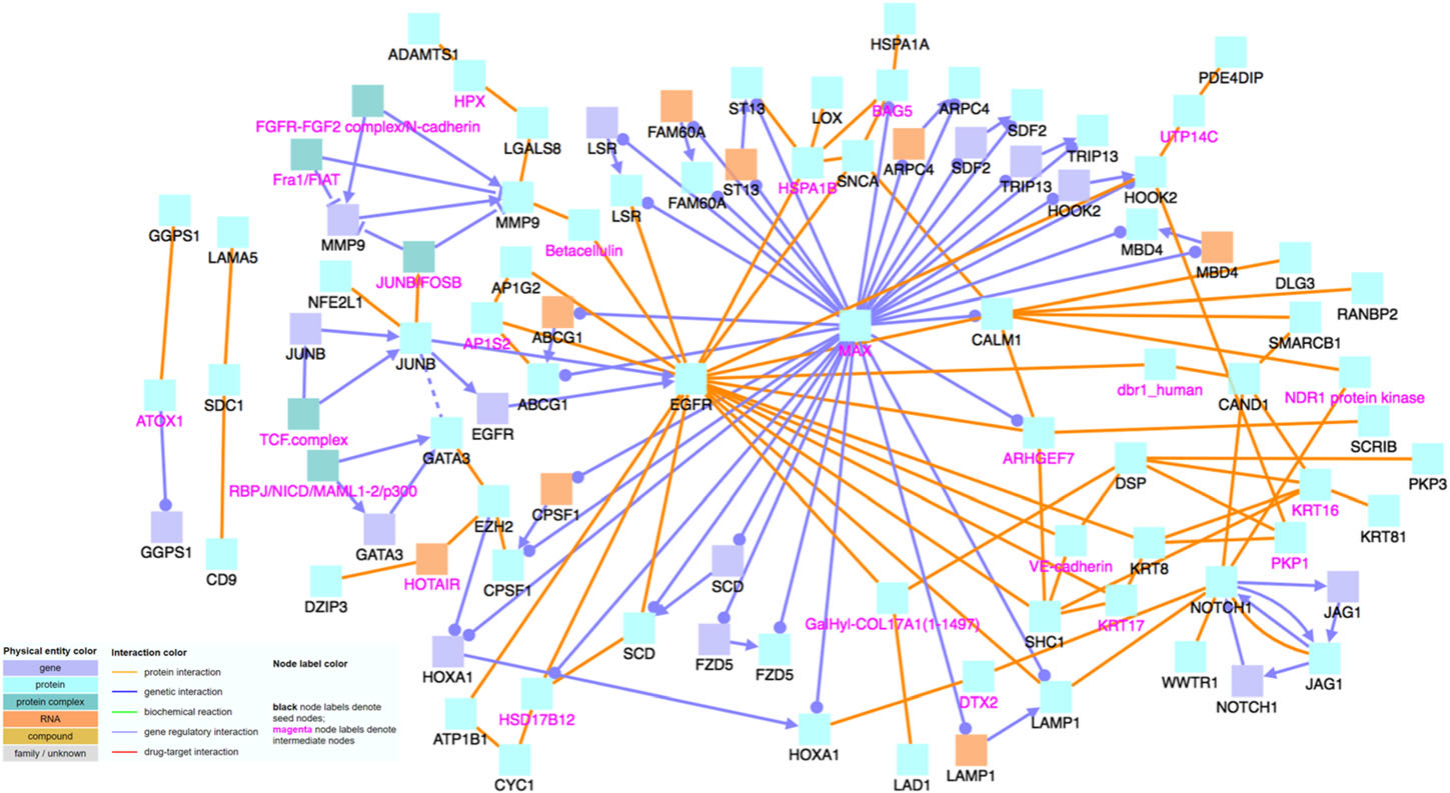
Network of 109 significant genes with positive correlation

**Fig. 2:**
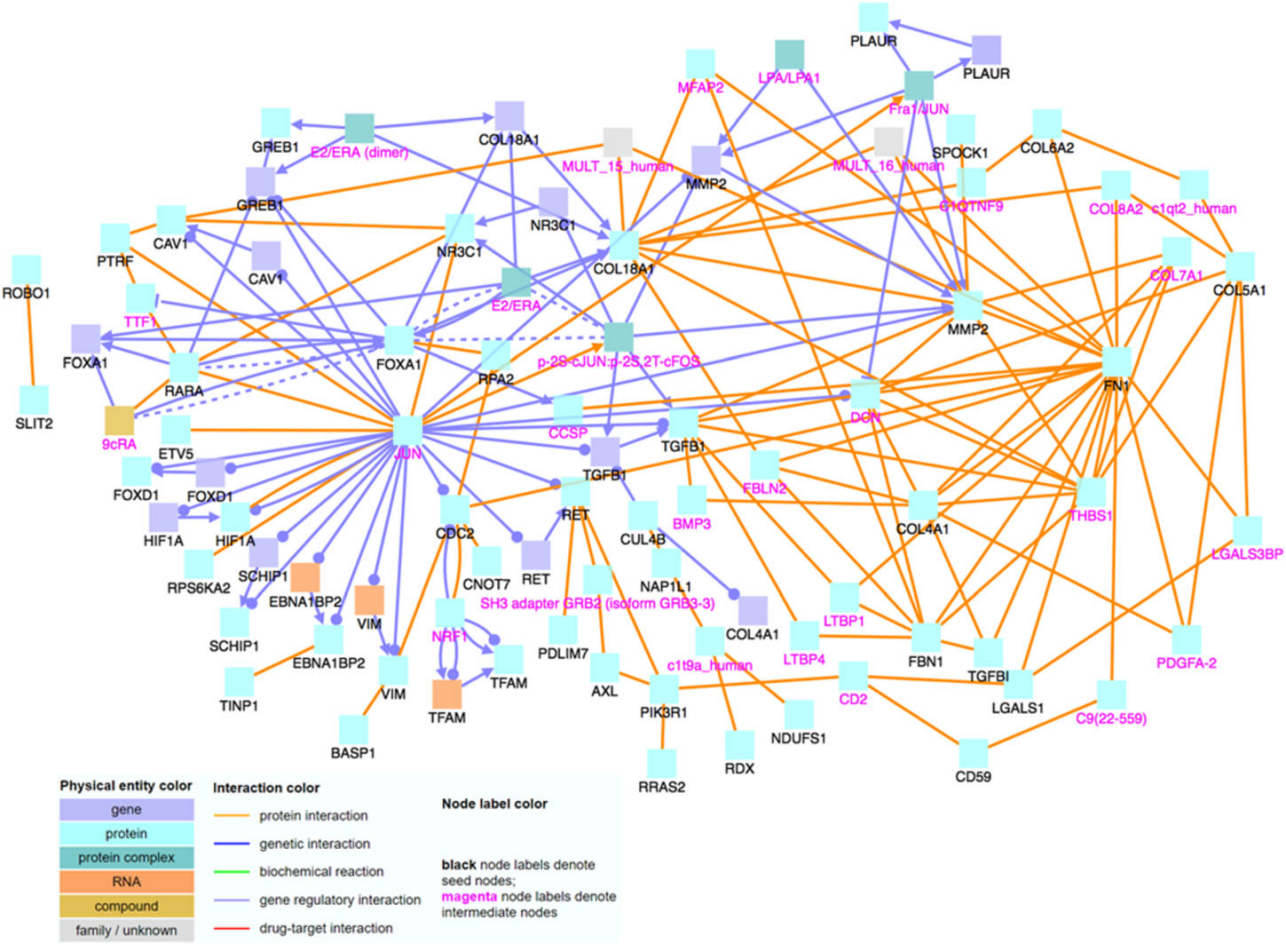
Network of 86 significant genes with negative correlation

**Fig. 3:**
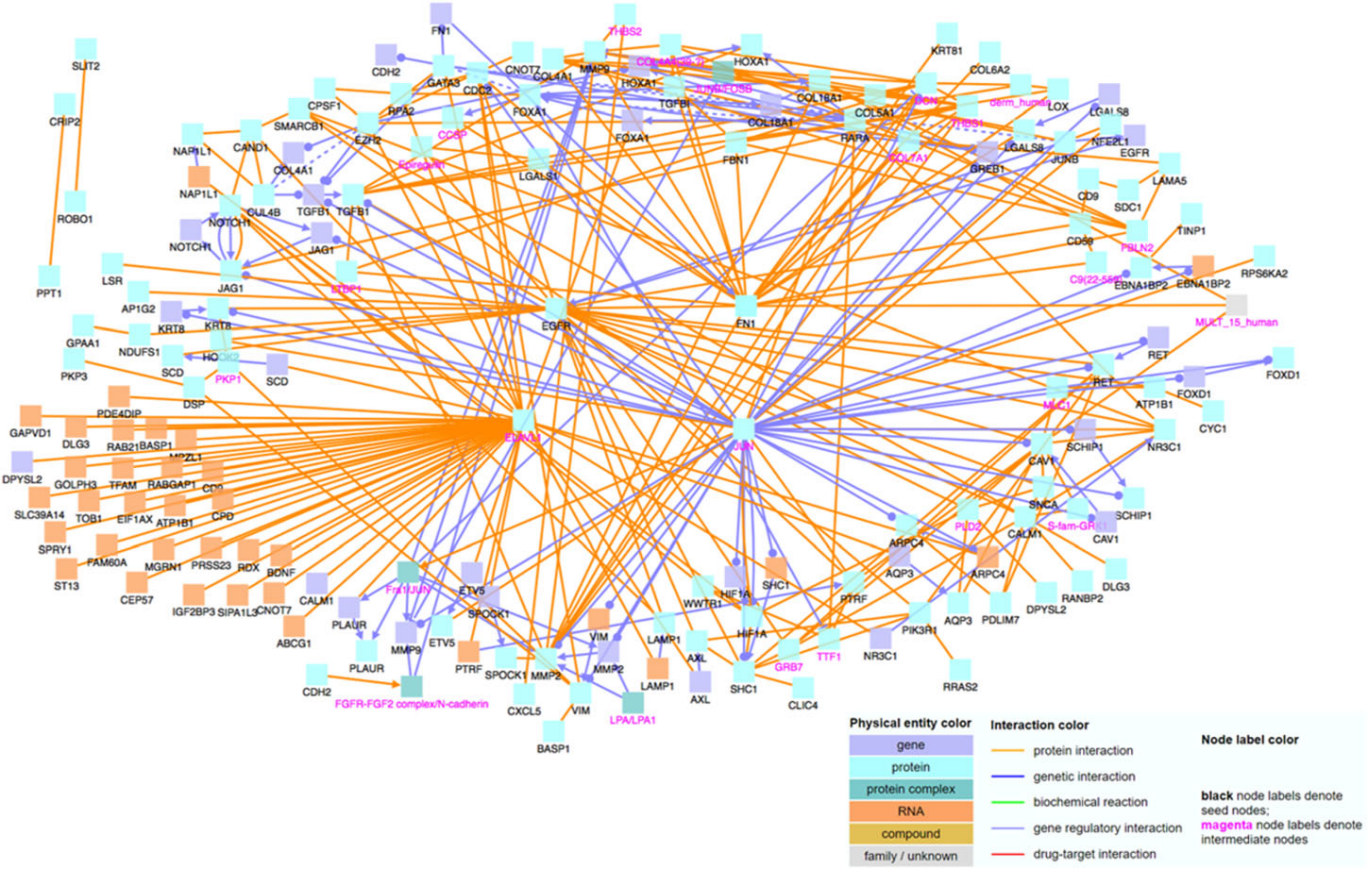
Network of 195 significant genes with positive and negative correlation

When analyzing the 109 positively correlated genes using CPDB, shown in Fig. 1, we notice that MAX and EGFR are major hub genes, genes that have a significantly large number of connections with other genes. Hub genes are important to study because changes in their expression level can cause major ramifications to a huge number of other genes and potentially disrupt major cell processes. Then, using the 86 negatively correlated genes, we create another induced network module, which is shown in Fig. 2. Similar to Fig. 1, there are several noticeable hub genes, namely: JUN, FN1, FOXA1, COL18A1, and MMP2. We also combine the list of 109 positively correlated and significant genes with the list of 86 negatively correlated and significant genes. We then use this list of 195 significant genes as an input file for CPDB. Figure 3 shows the CPDB analysis result, and as with both of the previous networks, there are several obvious hub genes: JUN, EGFR, ELAVL1, and FN1.

### Hub gene functions

Of the eight identified hub genes, JUN, MAX, and ELAVL1 are intermediate genes added by CPDB while the other five are significant genes we identified. The high connectivity of these genes, specifically to genes that we have identified as showing significant correlation between expression and methylation levels. This high connectivity suggests these hub genes may have a regulatory effect on related genes and are thus potential biomarkers.

GeneCards [17] is an integrative database that provides genomic, transcriptomic, genetic, and functional information on all annotated and predicted human genes. Using this database, we find that MAX regulates transcription and is linked to cell proliferation, differentiation, and apoptosis. These functions can all play a role in the formation of cancerous tumors.

Another hub gene EGFR is widely recognized for its important role in cancer [17]. Specifically, the amplification and mutation of EGFR proteins are shown to be driving events in basal-like breast cancers. EGFR is an epidermal growth factor receptor, a gene that is meant to check and control the growth of skin cells. In addition to being a hub gene, we also find that EGFR is a significantly methylated gene with a positive correlation between methylation and gene expression. If the methylation level for EGFR is low, the gene may be suppressed, which could lead to abnormal growth of skin cells. Conversely, high methylation levels would cause EGFR to be overexpressed and amplified, and as mentioned above, are driving events in cancer.

Additionally, we have identified FN1 as a hub gene. FN1 encodes fibronectin, a glycoprotein involved with migration processes, which includes metastasis [17]. Metastasis occurs often in the late stages of cancer and is where secondary tumors develop at a different location than the primary tumor. Thus, variations in the gene expression levels of FN1 due to methylation can strongly impact the spread of cancerous tumors.

FOXA1 is a transcription factor that is associated with ER+ breast cancer and luminal breast carcinoma [17]. It is involved with apoptosis and cell cycle regulation, among other functions. We find that FOXA1 has a significant negative correlation between methylation and gene expression. High methylation levels of this gene may cause low expression levels, which in combination with FOXA1’s functions of regulating the cell cycle and apoptosis, can lead to cancer.

Another hub gene is COL18A1. It encodes the alpha chain of type XVIII collagen, which is ultimately linked to the production of endostatin, a protein that is able to inhibit tumor growth [17]. Thus, COL18A1, when normally expressed, may aid in the prevention of cancer. However, we identify it as a gene with negative correlation between methylation and gene expression, so high methylation levels may suppress the expression of COL18A1, making our bodies more prone to the formation of cancerous tumors. The other three identified hub genes and their respective functions are summarized in Table 5.

**Table 5:** Functions of hub genes ELAVL1, JUN, and MMP2

Gene	Function
ELAVL1	Encodes a protein that contains several NA recognition motifs [17]. Selectively binds AU-rich elements in 3′ untranslated regions of mRNAs. Highly expressed in many cancers.
JUN	Encodes a protein that is highly similar to the avian sarcoma virus 17 protein and interacts directly with target DNA sequences to regulate gene expression [17]. It is mapped to 1p32-p31, a chromosomal region involved in both translocations and deletions in human malignancies.
MMP2	Protein coding gene with various functions that include tumor invasion and metastasis [17]. Also associated with estrogen receptor signaling.

### Further analysis of hub genes

**Fig. 4:**
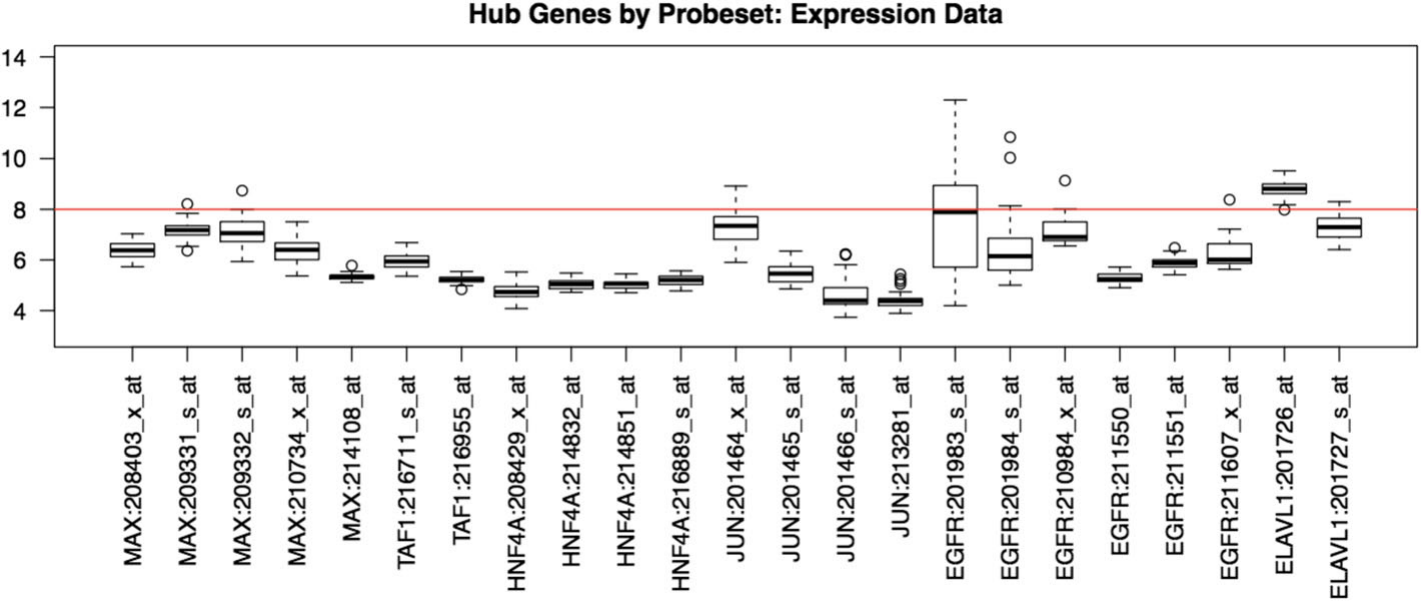
Gene expression levels of the identified hub genes

**Fig. 5:**
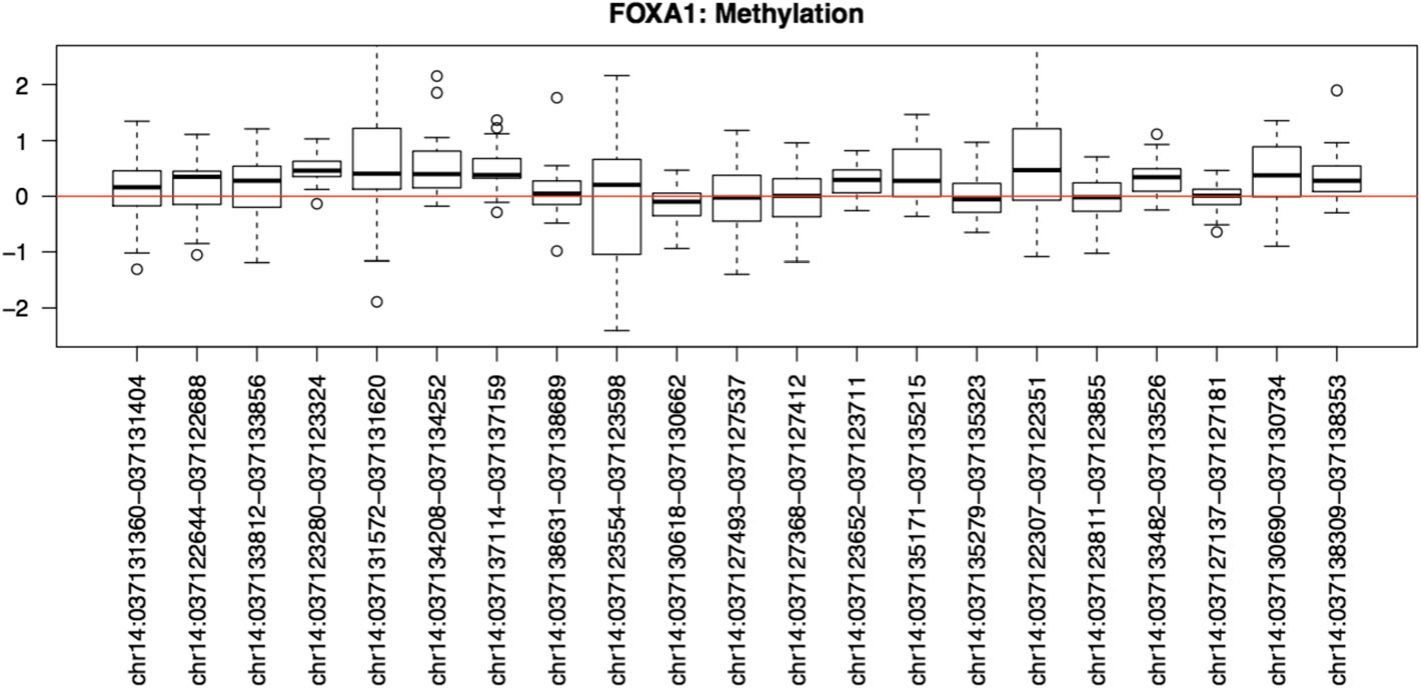
Methylation levels of hub gene FOXA1

We further study the characteristics of important hub genes using their gene expression and methylation levels. Upon finding results, we plot gene expression levels to help visualize and interpret our results. As shown in Fig. 4, we find that the identified hub genes tend to have relatively low gene expression levels, which are almost completely below 8 (indicated by the red line). As reference, housekeeping genes have gene expression levels at or above 8. Note, housekeeping genes carry out basic cellular functions and are well recognized for having steady gene expression levels. As mentioned above, we additionally study the methylation levels of these important hub genes. Plotting the methylation levels of hub genes by probe, we notice that these genes are in general hypermethylated. Figure 5 shows the methylation levels of one of the hub genes, FOXA1, and it demonstrates hypermethylation since the median methylation level of most probes is above zero.

We also run the t-test to determine whether each probe of a hub gene is hypermethylated or hypomethylated. To do so, we take the estimated mean methylation value and the *p*-value (or *p*). The estimated methylation level is the average methylation levels of all cell lines at each probe. We define a probe to be hypermethylated if the *mean methylation >* 0 and *p <* 0*.*05, conversely, we define a probe to be hypomethylated if the *mean methylation <* 0 and *p <* 0*.*05. If it fits into neither group, it is labeled as *N/A*. Table 6 shows that with the exception of ELAVL1, all the genes have more hypermethylated probes than hypomethylated and N/A probes combined. Additionally, if we only compare hypermethylated and hypomethylated probes, the gaps between the two are significantly more drastic.

**Table 6:** T-test results at the methylation microarray probe level for each gene

	# Hyper probes	% Hyper probes	# Hypo probes	% Hypo probes	# N/A	% N/A
MAX	11	68.75%	3	18.75%	2	12.50%
EGFR	11	52.38%	3	14.29%	7	33.33%
JUN	24	58.54%	2	4.88%	15	36.59%
FN1	8	80.00%	1	10.00%	1	10.00%
FOXA1	38	52.05%	13	17.80%	22	30.14%
MMP2	3	60.00%	1	20.00%	1	20.00%
ELAVL1	2	33.33%	2	33.33%	2	33.33%

### Brief summary and implication

Through studying the complex relationship between methylation and gene expression, we are able to elucidate some of the key characteristics of this relationship as well as identify eight hub genes. These genes (MAX, EGFR, ELAVL1, JUN, FN1, FOXA1, COL18A1, and MMP2) can be potential biomarkers. Discovering these biomarkers is a key step in identifying the cause and finding the cure for cancer. Although there have been previous similar studies, our integrative analyses provide more detailed evidence.

The depth of our research differentiates it from existing research. Sun et al. [8] similarly integrated gene expression and methylation data, but used only seven breast cancer cell lines and used summarized methylation data in each CpG island, which could be inaccurate because methylation signals may vary along a long CpG island. Our project goes further and examines 40 different breast cancer cell lines using microarray methylation data, we focus on exploring the effects of methylation on gene expression. Further, past research includes only the existence of a relationship between methylation and gene expression. However, our research goes several steps further by analyzing the genes with these specific characteristics, performing pathway analysis on them, and contextualizing these values. In this way, we have contributed a much better understanding of these possible epigenetic markers and their functions.

Through further research, we hope to find more hub genes by introducing more known information, readjusting the thresholds we used to determine significant genes, or adding different outside factors (apart from just ER status and tumor subtype). In addition, Chari et al.’s software package [22] shows the possibility of integrating more than just two types of data at once, which is another one of our future possible steps.

## Discussion

Gene expression microarray datasets are generated using the Affymetrix high-density oligonucleotide array human HG-U133A chip. We use the RMA algorithm to summarize the gene expression levels at the probeset level, which is a typical way of preprocessing Affymetrix gene expression array data. Methylation microarray datasets are generated using the Agilent 244K array at the probe level, which is designed for CpG site rich regions, i.e., CpG islands, not genes. In addition, because the methylation level across a CpG island varies, it is better to analyze the methylation data at the probe level to account for the variation. Therefore, we have developed our analysis methods based on the design and feature of these datasets.

Normality is a key assumption for linear models. For our gene expression data, when we randomly select 1000 probesets to conduct normality tests (Shapiro-Wilk normality tests), our test results show that, only about 8% of probesets have adjusted *p*-values < 0.05. This result is common for large and noisy microarray data; and it is also acceptable for our project because we have a relatively large sample size with *n* = 40 cell lines. When selecting significant genes, we exclude the probesets with normality test *p*-values < 0.05. That is, for the probesets related to the 195 significant genes, the normality assumption is valid.

Generally speaking, methylation signals in nearby probes are similar or correlated, and it is better to consider the correlation or dependence patterns in the analysis method as done by other researchers [23, 24]. Therefore, it is worth exploring deeper in this aspect, and we plan to work on this in our future projects. Next, we briefly explain why we did not include this pattern in our analysis. First, microarray data can be very noisy. When conducting the exploratory data analysis for our methylation signal data, we do not often see the nice correlation patterns in a relatively long region of a few hundred bases. Second, the length or size of each methylation probe is roughly 45 ~ 60 base pairs, and the median distance between two probes is about 50 bases, that is, from the beginning of the first probe to the beginning of next or second probe, the physical distance is about 100 bases; and from the first probe to the third probe it is about 200 bases. For very noisy methylation microarray data, there may not always be good correlation patterns within 100 ~ 200 bases. Third, we have checked the correlation of methylation signals between two consecutive probes; the majority of them have low correlation levels ranging from − 0.3 to 0.3. Probes near each other may have similar methylation patterns in some regions, but not necessarily in all genomic regions. The spatial dependence may vary from region to region in a whole genome. That is, the pattern is more complex than we currently know. Therefore, it is not proper to build one model for the whole genome in our data analysis. We plan to explore the spatial correlation patterns in more detail by conducting future research projects.

We define long and short genes based on the number of probesets (gene expression data) and probes (for methylation data) associated with each gene. In particular, we first summarize the numbers of probes and probesets and then select values around the third quartiles in these summaries to define long and short genes. Adjusting the cutoff values will give a relatively longer or shorter list of genes, but this does not affect the identification of hub genes very much. That is, the important hub genes that affect many other genes would still stand out in our analysis. For the analysis results presented in our paper, we choose the cutoff values that yield an optimal number of genes to study while maintaining the same results (i.e., same hub genes).

Regarding our method of gene-wise analysis, we choose to fit linear regression models for all pairs of probesets and probes before summarizing the results at the gene level, in order to maintain the most accuracy. If we summarize the methylation and gene expression data at the gene level first, we will lose a large amount of information. For example, in the gene expression data, two or more probesets of the same gene may display different expression levels. Similarly, probes associated with the same gene or CpG island in the methylation data may demonstrate very different methylation levels. Summarizing these datasets at the gene level before studying each pair of probeset and probe will prevent us from seeing and modeling the variation among the expression and methylation levels. By fitting linear models to each probeset-probe pair and studying long and short genes, we are able to conduct the most thorough and accurate analysis.

We have prepared for the Additional file 1 that includes detailed information for the 195 genes (109 with positive correlation and 86 with negative correlation). In this file, we have included columns to indicate if they are transcription factors or oncogenes. In addition, we have added columns that show the number of probes and probesets associated with each gene. We have compared these 195 genes with the known 100 hypomethylated and hypermethylated genes in primary breast cancer samples [25]. Among all the genes we identified, three of them have been reported in these 100 genes, they are CAV1, SLIT2, and ROBO1. In addition, using the breast carcinoma section of the public cancer database IntOGen (www.intogen.org), we have found that, out of the eight hub genes we identified, MAX, FN1, COL18A1, and EGFR are known driver genes. This means that specific mutations of these four genes can cause breast carcinomas. Furthermore, FOXA1 and FN1 cause the loss of function for genes; while MAX, EGFR, and MMP2 increase or change the activity or function of the targets. It is very likely that the other four hub genes we identified play a significant role, e.g., being a driver gene, but their driving functions have not been verified or reported yet.

## Conclusion

In this paper, we integrate the gene expression and methylation microarray data of 40 breast cancer cell lines to explore the complex relationship of the two, and their effects on breast cancer. We use linear models and perform analysis of variance to conduct the integrative analysis. By studying the coefficient of methylation as well as the *p*-value, we extract genes with significant correlation between methylation and gene expression. In our linear modeling, we have included other factors (ER status and tumor subtype) that may additionally play a role in over-expressing or suppressing gene expression. Investigating the genes using known databases, we find that several of these genes can significantly impact the formation of cancerous tumors, so we decide to study the interactions among these genes. Using CPDB, we map out the complex relationship among these desired genes and identify eight hub genes. We use the GeneCards database to find the functions of these hub genes, and we discover that they can all have an impact on cancer susceptibility. We then further study the methylation and gene expression of the hub genes and find that they have relatively high methylation levels and show signs of low or suppressed gene expression. Through our research, we have identified eight potential novel biomarkers: MAX, EGFR, ELAVL1, JUN, FN1, FOXA1, COL18A1, and MMP2. These potential novel biomarkers provide substantial contribution to breast cancer-related gene databases. Biomedical researchers may further study these genes to develop less expensive and more effective cancer screening and treatment processes. For example, researchers can perform DNA methylation analyses on patients and identify certain prevalent genes. Further, the identification of these genes may help other researchers find the cause of cancer on a genetic level and at the very least will allow for more detailed and narrower studies on important genes.

## Additional file

Additional file 1: Detailed information for the 195 significant genes. (XLSX 16 kb)

## Abbreviations

ANOVA: Analysis of variance
CPDB: ConsensusPathDB
CpG: Cytosine-guanine
ER: Estrogen receptor
MSigDB: Molecular Signatures Database
